# Dental anxiety among migraine patients

**DOI:** 10.25122/jml-2021-0004

**Published:** 2021

**Authors:** Yagoub Dhafer Alyami, Jana Khalid Farran, Jumanah Ateeq Alsubhi, Jehan Ahmed Omar, Nourah Abdulhadi Alsoubaia, Nourah Faisal Alyami, Joud Faisal Algoufi, Rawan Abdullah Alshehri

**Affiliations:** 1. Department of Oral and Maxillofacial Surgery, Vision College for Dentistry and Nursing , Jeddah, Kingdom of Saudi Arabia

**Keywords:** anxiety, depression, dental clinic, headache, migraine

## Abstract

In this study, we aimed to find a correlation between anxiety related to dental procedures and migraines. A cross-sectional study was performed on 171 patients who attended specific dental clinics. The patients were randomly categorized into a migraine group (83) and a control group. To determine the anxiety level, all the patients filled out a validated questionnaire (the Arabic version of the Modified Dental Anxiety Scale). All responses ranged from “not anxious” (scoring 1) to “extremely anxious” (scoring 5). Based on the patient responses, the total score was recorded and compared statistically between the two groups. The sound of drilling was one of the most vital factors causing anxiety and headaches in migraine patients. Comparing the presence or absence of headache and usage of analgesics between the two groups, migraine patients complained to have headaches during or after dental treatment more frequently than controls and used analgesics more than non-migraine controls. Migraine patients visiting dental clinics feel more anxious about the working environment and need certain modifications before, during, and after dental procedures.

## Introduction

Headache is commonly seen in individuals, tension headaches being commonest in the general population, while migraine headaches are commonly seen in patients seeking medical attention. Migraine is a common type of primary headache afflicting millions of people around the world with a wide variety of symptoms [1]. According to the International Headache Society (IHS), migraine headache causes a throbbing pain which is mainly unilateral and associated with photophobia, photophobia, nausea, and vomiting [2]. Studies revealed that around 12% of the American population suffer from migraine [3].

Anxiety is a common comorbid disorder seen in migraine patients [4]. Anxiety causes extreme and unnecessary worry that persists and is not restricted to a specific circumstance [5–7]. Dental anxiety is a commonly encountered problem in dentistry, leading to delay or avoidance of dental care [8]. A previous study by Farris *et al.* demonstrated the association between anxiety and migraine severity, especially in women [9]. To the authors’ knowledge, there is no study about the relation between migraine and dental anxiety, so we carried this study as the first one to find the correlation between dental anxiety and migraine in the Saudi population.

## Material and Methods

This is a cross-sectional study conducted at the Vision College, Jeddah, Saudi Arabia. All patients were informed about the study objectives and signed a consent form. The subjects were divided into two groups, the migraine group and the control group that was age- and sex-matched (non-migraine patients). The diagnosis of migraine was made by interviewing the patient and using the IHS diagnostic criteria for migraine [2]. To determine the anxiety level, all the patients filled out a validated questionnaire (Modified Dental Anxiety Scale – the Arabic version [8]. All responses ranged from “not anxious” (scoring 1) to “extremely anxious” (scoring 5). Based on the patient responses, the total score was taken as a sum of the individual score for each question, ranging from 5 to 25. Patients who scored 19 or above were considered to have high dental anxiety, possibly dentally phobic individuals.

The study objectives were to find out the relation between feeling anxiety when visiting the dentist and getting a headache in patients with migraine, to identify the factors from the dental work environment that increase the likelihood of getting a headache, to find out the probability of getting a headache during and after dentist visits, and to measure the usage of analgesics by the patients. With all these objectives, a questionnaire was developed based on the following questions:

1. Do patients feel anxiety when visiting the dentist’s clinic?
2. Is there any statistically significant difference in the feeling of anxiety between migraine and non-migraine patients?
3. What are the factors in the clinic environment (unit light, drilling sound, smell) that increase the feeling of headache in patients with migraine?
4. Is there any statistically significant difference in the probability of getting a headache during or after the dental treatment between those who suffer and do not suffer from migraine?
5. Is there any statistically significant difference in the degree of satisfaction of dental procedures between those who suffer and those who do not suffer from migraines?
6. Is there any statistically significant difference in the probability of taking analgesic medications before going to the dentist between those who suffer and those who do not suffer from migraines?
7. Is there any statistically significant difference in the feelings of migraine patients with anxiety attributed to the following variables:(sex, age, duration after migraine onset, the number of recurrences of the migraine per month and smoking)?

### Study sample

The study sample consisted of 171 patients who attended dental clinics associated with Vision College in Jeddah. The sample was randomized by means of a computer system; out of this sample, 83 suffered from migraine.

### Statistical methods

The Chi-Square test was used to calculate the difference between the frequency of individual responses, while the Mann-Whitney test was used to calculate the difference between the mean independent grade scoring. Kruskal-Wallis test was used to study the differences between the mean of several separate samples. The Statistical Package for the Social Sciences (SPSS) version 20 (IBM Corp. Released 2011. IBM SPSS Statistics for Windows, Version 20.0. Armonk, NY: USA, IBM Corp) was used for statistical analysis.

## Results

This is the first study that correlates anxiety related to dental environment and migraine. Regarding the answers to the first question, there was no statistical difference between the response given by individuals from both groups. The Chi-square value was not statistically significant (Table 1).

**Table 1.** Difference between the frequency of study sample responses to feeling anxiety (n=171).

**Feeling a high level of anxiety**	**Number**	**Percentage**	**Chi-Square value**	**Level of significance**
**Yes**	73	42.7%	3.66	0.06 Not Significant
**No**	98	57.3%		

Regarding question 2, we used the Chi-Square test for independent samples (cross-tabulation tables) to calculate the difference between the responses, and there was a statistically significant difference (at the 0.01 level) between the responses (Table 2).

**Table 2.** Chi-Square Test for independent samples and Cramer correlation factor when studying differences in anxiety because of the dentist attributable to migraine (n=171).

**Having headache**	**Feeling anxiety because of the dentist**		**Chi-square Value**	**Cramer’s factor**
	**Yes**	**No**		
**Migraine patients**	45 (26.3%)	38 (22.2%)	8.76	0.23
**Non-migraine patients**	28 (16.4%)	60 (35.1%)		

**Figure 1.**
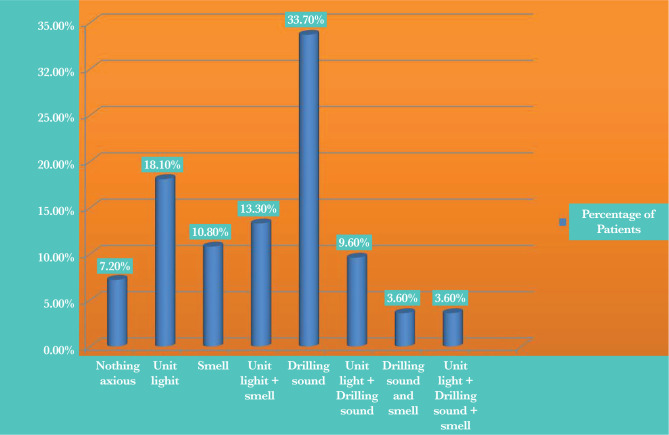
The percentage of migraine patients affected by factors found in the working environment of the clinics.

Regarding the third question about the factors in the clinic’s environment that increase migraine patients’ sense of headache, there was a statistical difference (at the level of 0.01; Table 3 and Figure 1) among the groups.

**Table 3.** Results of the Chi-Square test used to calculate the difference between the frequency of sample responses to the study on factors in the clinic's working environment (unit light/drilling sound/smell) that increase the feeling of migraine in patients with headaches (n=83).

**Factors in clinic working environment**	**Number**	**Percentage**	**Chi-square value**	**Level of evidence**
**Nothing anxious**	6	7.2%	45.01	0.01
**Unit light**	15	18.1%		
**Smell**	9	10.8%		
**Unit light + smell**	11	13.3%		
**Drilling sound**	28	33.7%		
**Unit light + drilling sound**	8	9.6%		
**Drilling sound and smell**	3	3.6%		
**Unit light + drilling sound + smell**	3	3.6%		

For question 4, we used the Mann-Whitney test to calculate the difference between the mean independent grade scoring. A statistical difference (at the 0.01 level) between migraine patients and those without migraine in respect to the degree of likelihood of headache during or after dental treatment was noted. We found that individuals that suffer from migraines are more likely to have headaches during or after dental treatment than those who do not have migraines (Table 4).

**Table 4.** Mann-Whitney test results when examining differences between patients who suffer and not from migraines in the likelihood of headaches during or after dental treatment.

**Getting headache**	**Numb Average grade er**	**Total grades**	**Total grades**	**(Z) Value**	**Level of evidence**
**Migraine patients**	83	100.83	8369	4.05	0.01
**Non-migraine patients**	88	72.01	6337		

Concerning the fifth question, we found a statistical difference (at the 0.01 level) between both groups. This indicates that individuals with migraines are more likely to feel comfortable during dental procedures than those who do not suffer from migraine (Table 5).

**Table 5.** Mann-Whitney test results when examining the differences between the mean grades of those who suffer and not from migraines in the degree of satisfaction of dental procedures.

**Getting headache**	**Numb Average grade er**	**Total grades**	**Total grades**	**(Z) Value**	**Level of evidence**
**Migraine patients**	83	105.05	8719.5	5.11	0.01
**Non-migraine patients**	88	68.03	5986.5		

Regarding the sixth question, we observed a statistical difference (at the 0.05 level) between the groups regarding the degree of analgesic intake before going to the dentist. This implies that individuals with migraines are more likely to take analgesics before going to a dentist than those who do not suffer from migraine (Table 6).

**Table 6.** Mann-Whitney Test results when examining the differences between the mean grades of those who suffer and not from migraines in the degree of likelihood of taking analgesic drugs before going to the dentist.

**Getting headache**	**Numb Average grade er**	**Total grades**	**Total grades**	**(Z) Value**	**Level of evidence**
**Migraine patients**	83	93.31	7744.5	2.28	0.05
**Non-migraine patients**	88	79.11	6961.5		

**Figure 2.**
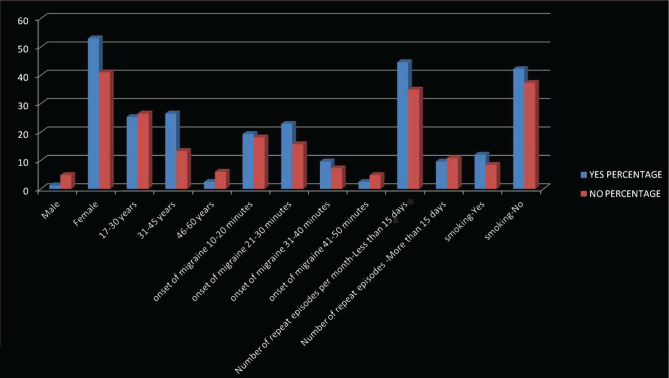
Differences in anxiety because of the dentist attributable to variables (sex, age, date of onset, frequency of disease per month, smoking).

Regarding question 7, there were no statistically significant differences between the groups related to anxiety that were attributable to sex, age onset of migraine, smoking and number of migraine episodes per month (Table 7 and Figure 2).

**Table 7.** Chi-Square test for independent samples (cross tabulation tables) when studying differences in the anxiety because of the dentist attributable to variables (sex, age, date of onset, frequency of disease per month, smoking) (n=83).

**Variables**	**Variable groups**	**Anxiety feeling**				P value
		**Yes**		**No**		
		**Number**	**Percentage**	**Number**	**Percentage**	
**Sex**	**Male**	1	1.2%	4	4.8%	2.51 NS
	**Female**	**44**	53%	34	41%	
**Age in years**	**17-30**	21	25.3%	22	26.5%	4.41 NS
	**31-45**	22	26.5%	11	13.3%	
	**46-60**	2	2.4%	5	6%	
**Onset of migraine in minutes**	**10-20**	16	19.3%	15	18.1%	1.53 NS
	**21-30**	19	22.9%	13	15.7%	
	**31-40**	8	9.6%	6	7.2%	
	**41-50**	2	2.4%	4	4.8%	
**Number of repeated sicknesses per month**	**Less than 15 days**	37	44.6%	29	34.9%	0.44 NS
	**More than 15 days**	8	9.6%	9	10.8%	
**Smoking**	**Yes**	10	12%	7	8.4%	0.18 NS
	**No**	35	42.2%	31	37.3%	

NS – Not Significant.

## Discussion

Regarding the question of whether patients feel anxiety when visiting the dental clinic, there was a convergence between the numbers and proportions of those who feel and those who do not feel anxiety when visiting the dental clinic. Our results contradict those obtained by Corah *et al.* that confirmed the feeling of anxiety [10].

There was a statistically significant difference between the groups in respect to feeling anxiety because of the dentist. This indicates that there is a difference in the feeling of anxiety attributed to migraine and hence the individuals with migraines are more likely to worry at the dentist. Most of the respondents revealed that the sound of drilling is one of the most important factors in the clinic’s working environment that increases migraine patients’ feelings of headache.

Individuals with migraines are more likely to have headaches during or after dental treatment than those who do not have migraines. Individuals with migraines are more likely to take analgesics before going to a dentist than those who do not have migraines. The absence of statistically significant differences in the feeling of anxiety caused by the dentist in migraine patients due to the following variables: sex, age, date of onset of the disease, number of recurrences of the disease per month, and smoking was noted. All these findings indicate the need to work with handpieces that make less noise. Soundproof earpieces could help reduce the possibility of getting headaches in patients who suffer from migraine. The clinics could use more dimmed lighting; also, placing the waiting areas far away from the operating rooms could help to help reduce stress, which helps lower migraine triggers. Moreover, well-ventilated operating rooms and waiting areas could reduce the smell that usually is a migraine trigger.

### Limitations of the study

The present study had some limitations; firstly, the accuracy of patients’ answers to the questionnaire could have been influenced by their embarrassment. Secondly, the number of females was larger compared to males. The third limitation was that the participants were selected only from one site, namely the Vision College, Private Clinics.

## Conclusion

We found out that there is a significant difference in the anxiety levels of migraine patients regarding dental treatment, and there is a need to include migraine in the history taking prior to treatment procedures and modify the dental work environment accordingly. However, we recommend expanding the research domain to several clinics and collect more samples all around the community, increase the number of male participants, and consider the inclusion of more diverse age groups.

## Acknowledgments

### Ethical approval

The approval for this study was obtained from the Ethics Committee of the Vision College for Dentistry and Nursing , Jeddah, Kingdom of Saudi Arabia (approval ID: 19-10/3).

### Consent to participate

The participants gave written informed consent for participation in this study.

### Conflict of interest

The authors declare that there is no conflict of interest.
